# Metformin versus Insulin in the Management of Pre-Gestational Diabetes Mellitus in Pregnancy and Gestational Diabetes Mellitus at the Korle Bu Teaching Hospital: A Randomized Clinical Trial

**DOI:** 10.1371/journal.pone.0125712

**Published:** 2015-05-06

**Authors:** Titus Beyuo, Samuel Amenyi Obed, Kenneth Kweku Adjepong-Yamoah, Kwasi Agyei Bugyei, Samuel Antwi Oppong, Kissinger Marfoh

**Affiliations:** 1 Department of Obstetrics and Gynecology, Korle Bu Teaching Hospital, Accra, Ghana; 2 Department of Medicine and Therapeutics, Korle Bu Teaching Hospital and Department of Pharmacology, University of Ghana Medical School, Accra, Ghana; 3 Department of Pharmacology, University of Ghana Medical School, Accra, Ghana; 4 Public Health Unit, Korle Bu Teaching Hospital, Accra, Ghana; University, ITALY

## Abstract

**Objective:**

To determine if metformin monotherapy or metformin in combination with insulin is equally effective as insulin monotherapy at glycemic control in diabetes mellitus in pregnancy among Ghanaians.

**Methods:**

This was a study involving 104 pregnant women with type 2 diabetes mellitus (T2DM) or gestational diabetes mellitus (GDM) at 20-30 weeks gestation. Participants were randomized into metformin and insulin treatment groups. Starting dose of metformin was 500 mg once a day and increased gradually over two (2) weeks, to meet glycemic targets. Insulin was added if targets could not be reached on metformin alone at maximum doses. Total daily dose of premixed insulin at initiation was calculated as 0.3 IU/kg body weight and titrated upwards to achieve glycemic control. Glycemic profile monitoring was done every two weeks.

**Results:**

The two hour post prandial blood glucose (2HPG) levels were significantly lower in the metformin group than the insulin group (p= 0.004).

**Conclusion:**

The findings of this study suggest that metformin monotherapy is effective in achieving glycemic targets in the management of diabetes in pregnancy. It is more effective than insulin in lowering the 2HPG level.

**Trial Registration:**

Australian New Zealand Clinical Trials Registry (ANZCTR) ACTRN12614000942651

## Introduction

Diabetes mellitus (DM) is a metabolic disorder of multiple etiology characterized by, chronic hyperglycemia with disturbance of carbohydrate, fat and protein metabolism resulting from defects in insulin secretion, insulin action, or both [1]. The World Health Organizations (WHO) recommends that Gestational diabetes mellitus (GDM) be diagnosed when hyperglycemia is noticed for the first time at any stage of the pregnancy with one or more of these abnormal values on 75 g oral glucose tolerance test- fasting plasma glucose 5.1–6.9 mmol/l, 1-hour plasma glucose 10.0 mmol/l or 2- hour plasma glucose 8.5–11.0 mmol/l. [2]. The prevalence of diabetes mellitus world- wide is projected to increase [3].

There is no national data on the prevalence of DM among pregnant women in Ghana. Data from Greater Accra estimates the crude prevalence of diabetes mellitus among the adult population as 6.3% [4]. The use of insulin has traditionally been the main stay in the management of DM in pregnancy not adequately controlled on diet and exercise. Though effective, the use of insulin is associated with some disadvantages such as the inconvenience of repeated injections, high cost, storage problems and hypoglycemia. In one Indian study the cost of insulin was found to be ten-fold higher than that of metformin [5].

Metformin is a promising oral agent being widely used for this indication in a number of countries. There is evidence that metformin is an effective oral hypoglycemic agent that improves insulin sensitivity [6]. Metformin has been shown not to be inferior to insulin for glycemic control in GDM [7, 8, 9]. Despite these findings, data on randomized controlled trials involving metformin and insulin in the management of Type 2 Diabetes Mellitus (T2DM) in pregnancy and GDM are lacking in the indigenous African population. The pharmacogenetics of metformin does not support extrapolation of research findings from one population to another. The excretion of metformin is influenced significantly by a variant allele (*SLC22A2*) and could result in treatment failure or toxicity in carriers of this allele [10], hence the need for studies in our population.

## Materials and Methods

The protocol for this trial and supporting CONSORT checklist are available as supporting information; see S1 Checklist and S1 Protocol. We conducted a randomized, open-label trial designed with intention—to-treat analysis at the Maternity Unit and the Diabetes Centre of the Korle Bu Teaching Hospital. The study involved both in-patients and out-patients recruited between 1^st^ January, 2013 and 31^st^ October, 2013. For a set of four patients seen at the clinic for the first time, they were made to ballot by picking randomly one paper with an inscription each from an opaque envelope. This assigned participants to one of the two treatment groups—insulin and metformin. The sequence of picking was in the order in which they reported to the clinic; “first to report, first to pick”.

Women aged 18 to 45 years who were pregnant with single fetus at gestational age of 20 to 30 weeks and have been diagnosed with T2DM or GDM and met the Hospital’s criteria for starting insulin therapy were considered eligible subjects. The gestational age of 20 weeks was conveniently selected as the entry point because at the study sites, it is from this gestation that women are organized into an antenatal clinic with scheduled visits. Women, who were found not to be diabetic at 20 weeks gestation, had the 75 g oral glucose tolerance test (OGTT) repeated at 28 weeks gestation. Those who were diagnosed at this gestation were included in the study and those who tested negative were excluded with no further testing. Women previously diagnosed with T2DM and on therapy were excluded from OGTT.

A diagnosis of pre-gestational diabetes mellitus was made when a plasma glucose concentration is **≥** 7 mmol/l after an overnight fast or plasma glucose concentration is **≥** 11.1 mmol/l 2 hours after a 75g glucose drink [2]. We adopted the ADA 2012 recommendation for diagnosing GDM which are FBS > 5.1 mmol/l; 1HPG >10.0 mmol/l or 2HPG >8.5 mmol/l. Diagnosis is made when one or more of these values are exceeded [11]. Newly diagnosed clients were managed on diet and exercise and when glycemic control were unsatisfactory, they were then recruited into the study and put on the treatment protocol.

Exclusion criteria included patients with Type 1 Diabetes Mellitus and T2DM who have previously failed to achieve glycemic control on metformin monotherapy. Patients with allergies to metformin were also excluded.

Using the two sampled mean formula [12] and assumed standard deviations of 6.2 ±0.6 mmol/l and 6.4±0.9 mmol/l in the metformin and insulin groups respectively from a previous study [7] the estimated sample size was 47 per group. We recruited 52 in each group to allow for a 10% non-respondent rate. From pre-trial estimates the minimum difference in mean 2HPG levels between the two groups of 4mg/dl (0.22 mmol/l) is required to give a power of 80% to the study at a significance level of α = 0.05 (two sided). This difference was assumed to be clinically significant.

In the metformin group, starting dose of metformin was 500 mg once a day and increased gradually over two weeks. The maximum dose allowed per study protocol was 2500 mg per day. Insulin was added if targets could not be reached on metformin alone at maximum doses. Treatment glycemic targets of FBS < 5.5 mmol/L and 2HPG < 7.0mmol/L recommended by Australian Diabetes in Pregnancy Society [13] were selected for the study.

In the Insulin group both soluble insulin and premixed insulin were prescribed. There was no brand restriction. Both premixed insulin and soluble insulin were administered subcutaneously in the deltoid region. Total daily dose of premixed insulin at initiation was calculated for most patients as 0.3 IU/kg body weight. However for patients admitted with high blood glucose levels and managed on sliding scale with soluble insulin their starting doses were based on total daily requirement. The total daily dose was then divided into two: two-thirds of the dose was given in the morning 30 minutes before breakfast and one-third of the daily dose given in the evening 30 minutes before supper. The total dose of insulin was titrated for each patient to achieve the above glycemic targets. Few patients combined both soluble insulin administered three times a day before meals with premixed insulin on regular basis to achieve glycemic control targets

Patients who did not achieve glycemic targets on their out-patient doses after two attempts at titrations were admitted to the ward and treated with soluble insulin to determine their new optimum insulin requirements. All patients were educated by both nurses and doctors while on admission on the disease and self-administration of the correct doses of insulin before discharge.

Basic demographic data were recorded. Subjects were followed through their index pregnancy, with blood glucose and maternal weight monitoring every 2 weeks. The dose of metformin or insulin required for optimal glycemic control for each patient and peri-partum events like gestational age at delivery, type of delivery, fetal birth weight, and Neonatal Intensive Care Unit (NICU) admissions were retrieved from patients notes and analyzed. All patients were weighed.

Daily blood glucose self-monitoring was encouraged. The values used in the analysis were that of the laboratory results from the Diabetes Research Laboratory. All laboratory samples were analyzed at the Diabetes Research Laboratory. Venous blood was used for laboratory blood glucose profile analysis. Analysis of Fasting Blood Glucose (FBG), one- hour postprandial glucose (1HPG), two- hour postprandial glucose (2HPG) was done using a chemistry analyzer. The method for glucose determination was enzymatic photometric testing. Glycemic profile measurement did not include glycosylated hemoglobin (HbA1c) levels for logistic reasons.

All statistical analyses were done using statistical package for social science (SPSS) version 16.0. Data were examined for assumptions of normality (i.e. Shiparo-Wilk) and homogeneity of variance (i.e. Levene’s test). Mean between two groups were compared using t test and difference in proportions between two groups using chi square test. Differences in FBG, 1HPG and 2HPG were determined by mixed design repeated measure ANOVA with 3 within subject factors and one between group factors. All the assumptions were met except the sphericity. Thus, the Greenhouse-Guisser correction was used in the analysis. If difference was detected a post-hoc analysis was done using the t tests to examine intragroup difference and intergroup comparisons. Bonferoni test was used to adjust for the p-values. A p—value <0.05 was considered significant.

### Ethics statement

All subjects gave written consent before enrolment and patients who did not give consent were excluded. The study was approved by the Ethical and Protocol Review committee of the University of Ghana Medical School (protocol number: MS-Et/M.4 —P3.3/2012-13) on the 21^st^ December 2012 before enrolments of participants from 1^st^ January, 2013 to 31^st^ October 2013. Patient follow up was completed by 15^th^ February 2014. The trial was registered retrospectively with the Australia New Zealand clinical trial registry (ACTRN12614000942651). The delay in registering the trial was due to financial constraints. The authors confirm that all ongoing and related trials for this drug/intervention are registered.

## Results

**Fig 1:**
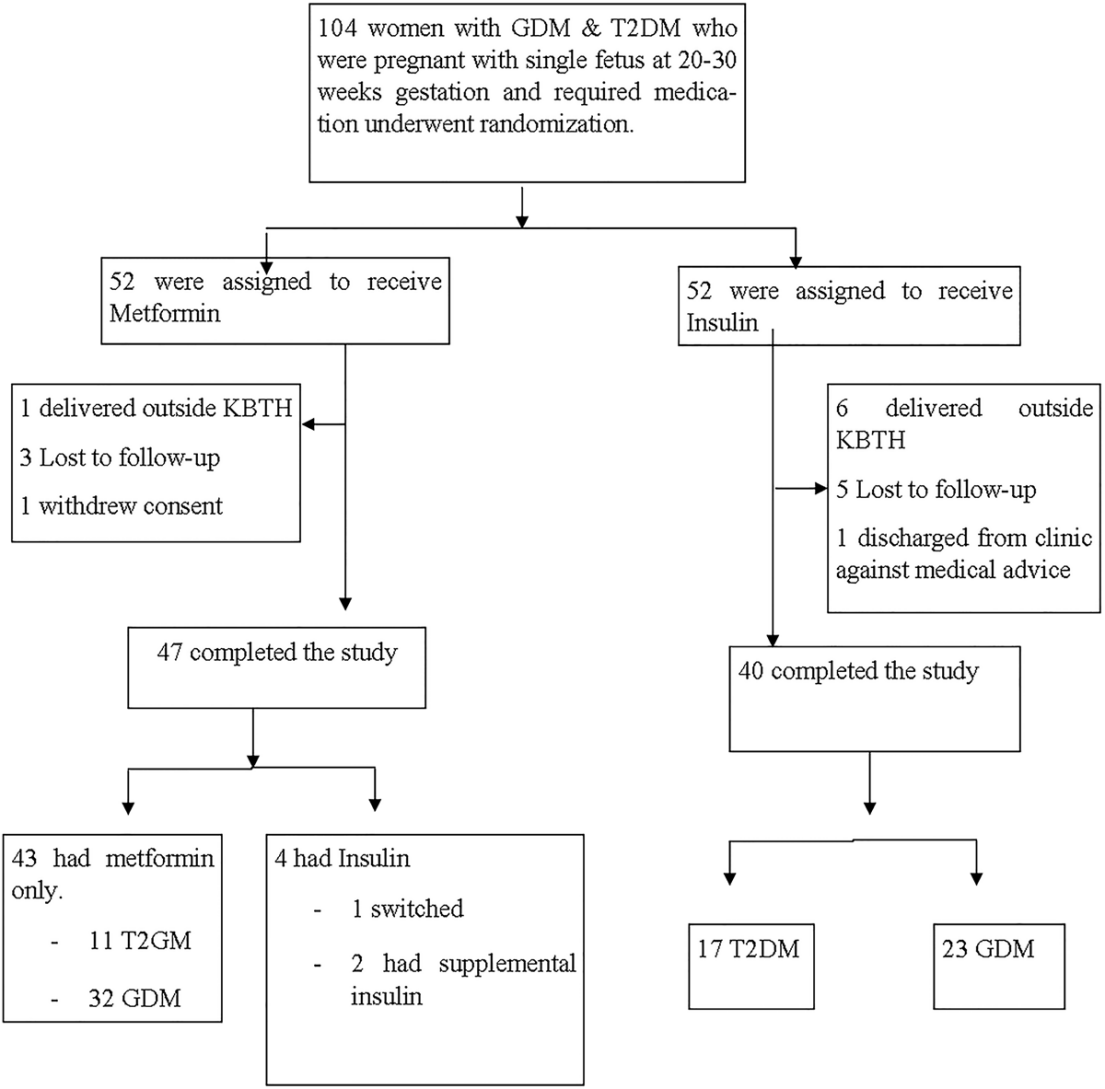
Flow diagram of participants from enrolment to term.

A total of 104 participants with GDM & T2DM were enrolled in the study. Ninety percent and 76% of participants in the metformin and insulin group respectively completed the study. Four participants in the metformin group also received insulin (Fig 1).

The two treatment groups were comparable in several demographic and pre-enrolment characteristics (Table 1). There were no significant differences observed in age, parity, marital status, BMI and the two pre- treatment weight categories. There was, however, a significant difference in the gestational age at enrolment with the metformin group being recruited at a higher gestational age, p = 0.017. No significant difference was observed in the classification of diabetes between the two treatment groups. Again, no significant difference was observed in the prevalence of co-morbid conditions in the index pregnancy. The obstetric history was similar in both treatment groups.

**Table 1:** Demographic characteristics of participants.

Variable	Metformin (N = 43)	Insulin (N = 40)	p-value
Age-yr (mean ±SD)	33.51 ±4.67	33.10±4.56	0.686
**Parity {no. (%)}**			
Nulliparous	7 (16.3)	8 (20.0)	0.897
P1–P4	33 (76.7)	29 (72.5)	
≥P5	3 (7.0)	3 (7.5)	
**Marital Status {no. (%)}**			
Married	43 (100.0)	39 (97.5)	0.482
Single/Divorced	0 (0.0)	1 (2.5)	
BMI-kg/m^2^	33.47± 6.95	32.61±6.21	0.56
**Weight at enrolment {no. (%)}**			
<90kg	24 (57.1)	28 (70.0)	0.227
≥90kg	18 (42.9)	12 (30.0)	
**Gestational Age** (weeks) {median, inter-quartile range (25–75)}	28, (26–29)	26, (23–28)	0.017
**Classification of diabetes {no. (%)}**			
GDM	32(74.4)	23(57.5)	0.103
T2DM	11(25.6)	17(42.5)	
**Co-morbid condition in pregnancy {no. (%)}**			
Essential Hypertension	6(14.0)	5(12.5)	1.000
Sickle cell disease	1 (2.3)	0 (0.0)	1.000
Others*	3 (7.0)	3 (7.5)	1.000
**Obstetric history {no. (%)}**			
Miscarriage	12 (27.9)	15 (37.5)	0.351
Stillbirths	3 (7.0)	2 (5.0)	1.000
Early neonatal deaths	1 (2.3)	2 (5.0)	0.607
Big Baby (>4.0kg)	5 (11.6)	2 (5.0)	0.095
Caesarian section	14 (32.6)	14 (35.0)	0.820
Others**	3 (7.0)	3 (7.5)	1.000

*One patient each in the metformin group had malaria, multiple uterine fibroid, anemia as a co-morbid condition whiles one each for insulin had hepatitis B, G6PD and goiter

**One patient each in the metformin group had intrauterine fetal demise (IUFD), gestational diabetes, and gestational hypertension in their obstetric history whiles insulin group had one patient each with gestational diabetes, post-partum hemorrhage and IUFD.

Two subjects who were randomized to receive metformin had supplemental insulin because of difficulty in achieving glycemic targets at maximum doses. This number was considered too small for separate comparative analysis with respect to doses of medications with either treatment groups. They were, however, analyzed as part of the metformin treatment arm.

**Fig 2:**
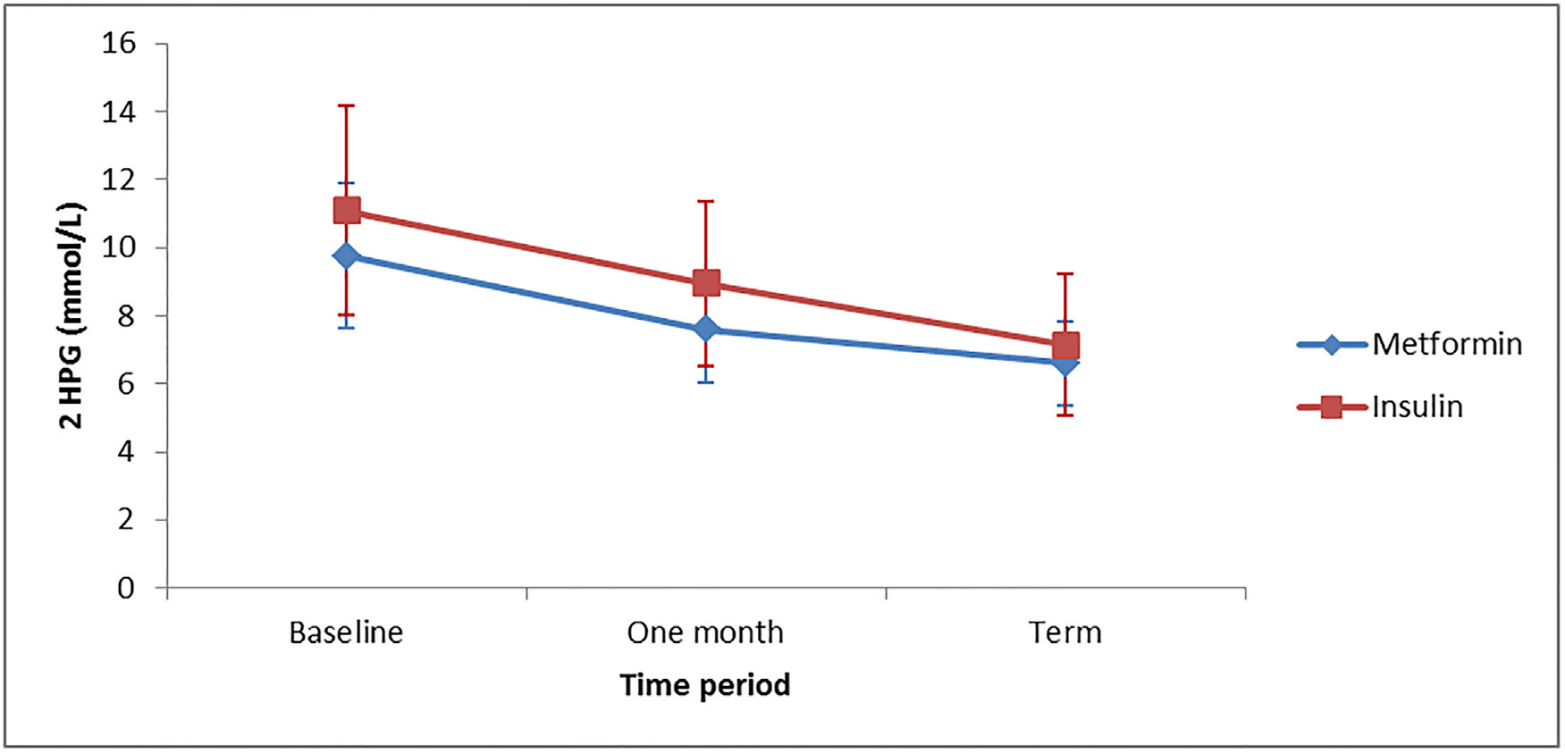
Mean 2HPG levels at baseline, one month from enrolment and at term for the two treatment groups.

The primary outcome of this study, the 2HPG levels were significantly lower in the metformin group compared to the insulin group with p value of 0.004. The 2HPG level decreased from baseline to term for both treatment groups (Fig 2). A repeated measure analysis of variance, using the Greenhouser-Guisser correction, showed that there were significant differences in the 2HPG values between the different times of measurement (*F* (2, 78) = 64.26, *p* < .0001). No significant difference in 2HPG was found between the insulin and metformin group with respect to time, (*F* (2, 78) = 3.031, *p* = 0.054). However, the metformin group had a significant 1.21mol/L drop in 2HPG compared to insulin (*F* (1, 79) = 9.169, *p* = 0.003). Secondary outcome measures included FBG, 1HPG, maternal weight gain, pregnancy outcome and feto-neonatal outcomes. Only the glycemic control is discussed in this publication.

**Fig 3:**
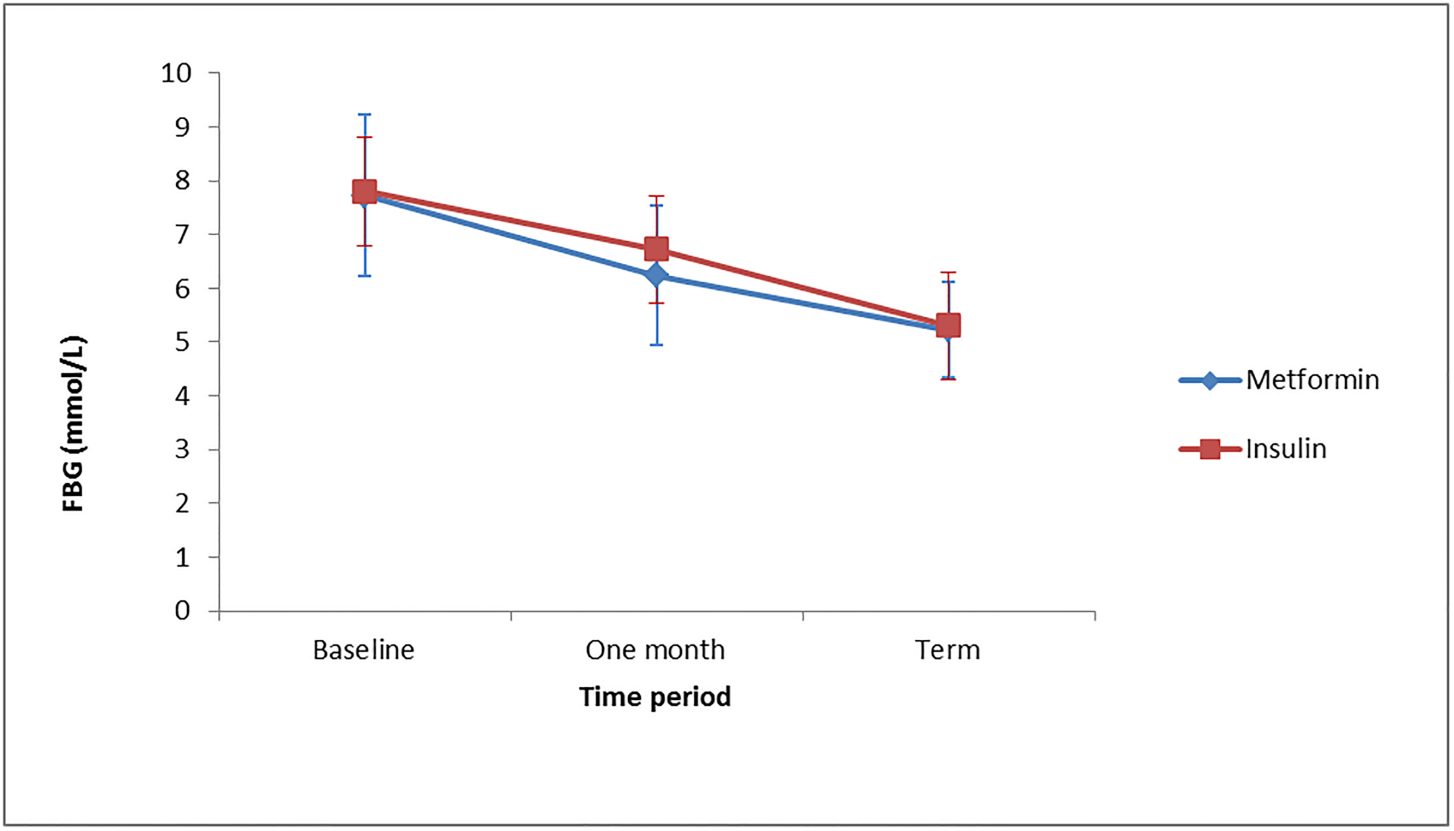
Mean FBG levels at baseline, one month from enrolment and at term for the two treatment groups.

**Fig 4:**
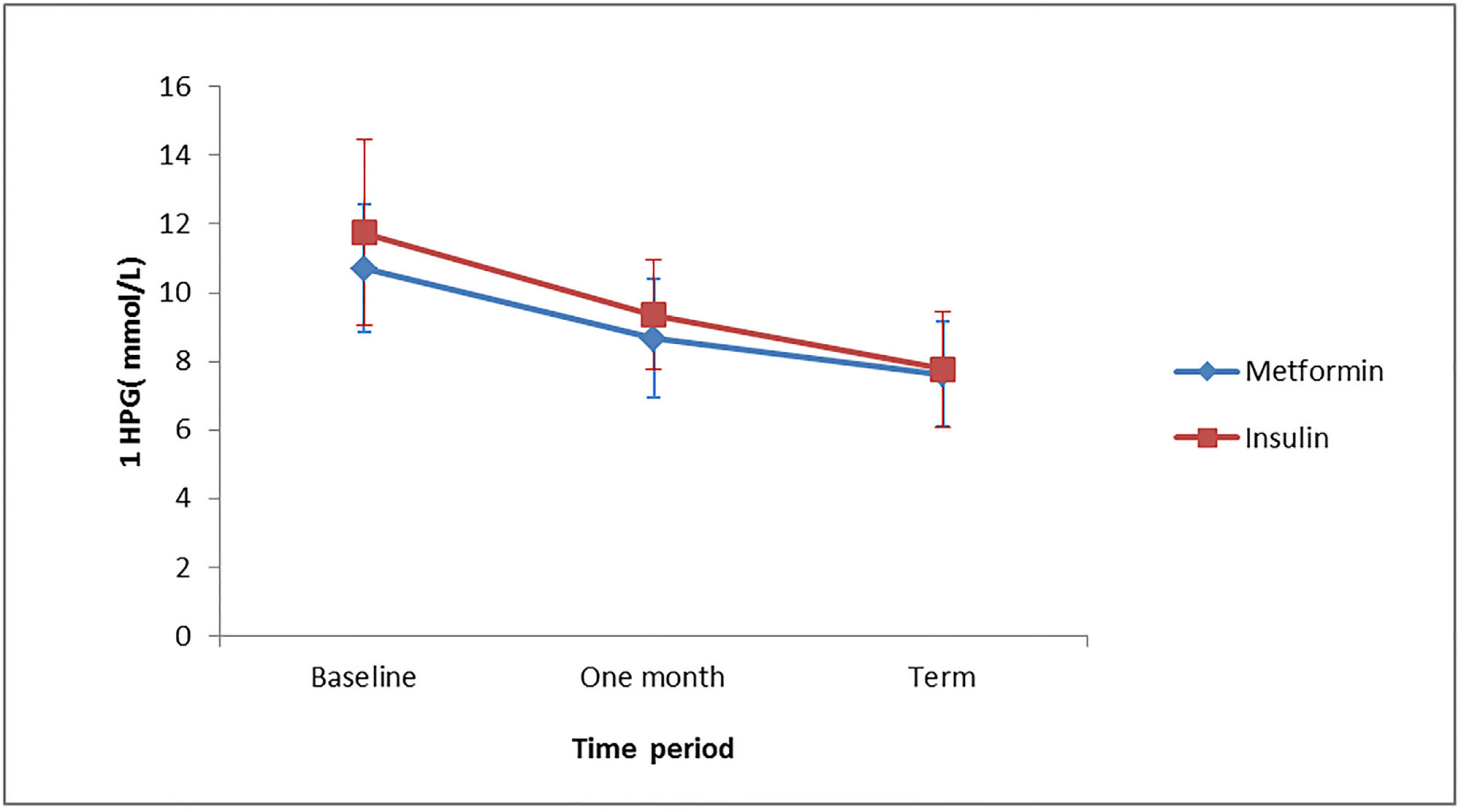
Mean 1HPG levels at baseline, one month from enrolment and at term for the two treatment groups.

The mean FBG and 1HPG level from enrolment to term was similar in both treatment groups with p values of 0.928 and 0.078 respectively. The FBG level decreased from enrolment to term in both groups (Fig 3). The mean FBG for metformin remained lower than that of insulin in the course of the study but they approximate at the exit point, but no significant difference was observed. A similar trend was observed for 1HPG (Fig 4).

The median dose of medication in both groups increased from enrolment to term. In the metformin group the median dose increased from 1000 mg daily in the first month of enrolment to 2000mg at term. In the insulin treatment group the median daily insulin requirement increased from 30 IU to 43 IU.

## Discussion

A key objective of this study was to determine blood glucose profile in pregnant women with GDM or T2DM who were treated with metformin on one arm and compare with the profile of women treated with insulin on the other arm. The 2HPG levels were significantly lower in the metformin group than the insulin group. This is consistent with results of a larger trial involving GDM patients only [7]. Further analysis of the pattern of glycemic control with repeated measure analysis of variance showed that there were significant differences in the 2HPG values between the different times of measurement (baseline, one month, term). No significant difference in 2HPG was found between the insulin and metformin group with respect to time as both groups had a reduction from baseline to term. However, the metformin group had a significant 1.21 mmol/L drop in 2HPG compared to insulin after a month of treatment. This reduction was larger than our pre-trial estimate of 0.22 mmol/L

We found the mean FBG and 1HPG level from enrolment to term were similar in both treatment groups (Table 2). This agreed favorably with results from a large randomized controlled trial involving only GDM patients [7]. The FBG level decreased from enrolment to term in both groups. The FBG levels for the two treatment groups were comparable at baseline with that for metformin being slightly lower than that of insulin, but they approximate at the exit point. The mean 1HPG values showed a decreasing trend in both treatment groups. No significant difference was observed at all times of measurements between the two groups. We found metformin to exhibit a more pronounced blood glucose lowering effect during the 2-hour post prandial period than the 1-hour post prandial period. This observation needs further research. The proportion of pre-gestational diabetes mellitus (T2DM) among pregnant women with diabetes who were enrolled in the study was 33.73%. It is similar to another report from the West African sub region [14]. This suggests that the contribution of pre-gestational diabetes to the overall burden of diabetes in pregnancy is significant. It has been suggested that prevalence of Type 2 diabetes mellitus is increasing in the young and reproductive age group [15]. Caregivers must be aware of this sub-group and be equipped with the knowledge to optimize preconception and as well as antenatal glycemic control to ensure good outcome for mother and baby.

**Table 2:** Mean glycemic profile from enrolment till term.

	Metformin	Insulin	P-value
**FBG (mmol/L)**	6.42 ±0.98	6.62 ±1.57	0.928
**1HPG (mmol/L)**	8.95 ±1.27	9.62 ±1.44	0.078
**2HPG(mmol/L)**	7.84 ±1.43	9.05 ±1.89	0.004

The gestational age at which GDM is diagnosed is very important. This is because women, in whom diagnosis of diabetes mellitus is made for the first time in the first half of pregnancy, invariably become diabetic after the pregnancy. They have a higher incidence of obstetric complications, recurrent GDM in subsequent pregnancies, and future development of Type 2 diabetes [3]. In our study, the gestational age at enrolment may not exactly represent the gestational age at diagnosis in most cases. This is because for participants with GDM, following diagnosis most are first managed on diet and exercise before enrolment if glycemic control is not satisfactory. There could also be delays in presentation, diagnosis and referral from peripheral health facilities to the study sites.

The median dose of medication in both groups increased from enrolment to term. This may be a reflection of the falling insulin sensitivity with advancing gestational age [15].

One objective of the study was to estimate the supplemental insulin requirement for patients in the metformin arm who may require insulin for effective glucose control. Only two subjects who were randomized to receive metformin had supplemental insulin because of difficulty in achieving glycemic targets at maximum doses. This number was considered too small. Therefore comparative analysis with respect to dose with either treatment groups was not done. It has however been shown that patients on metformin requiring supplemental insulin use smaller doses compared to those managed on insulin only [7]. Two subjects switched from metformin to insulin inadvertently due to clinician errors.

The response of our study population to metformin was similar to that from a predominantly European or white population [7].

The use of both T2DM and GDM patients is a major strength for this study as it reflects real life situation. Most studies have focused on GDM only.

The lack of blinding is a limitation of this study, however, the differences in the physical properties as well as the route of administration made blinding difficult. This could have led to over estimation of the effect of metformin. Subjects in the metformin arm preferred metformin because it is taken orally and did not desire to be switched to the insulin arm. Another limitation to this study is the high attrition rate in the insulin arm of the study.

## Conclusions

The findings of this study suggest that metformin monotherapy may be equally effective in achieving glycemic targets in the management of diabetes in pregnancy. It is more effective than insulin in lowering the 2HPG level

We recommend that large sample sized randomized controlled trials be conducted with long follow up to fully evaluate the long term effects of metformin therapy on both offspring and mother. A larger study could also evaluate the compliance and cost benefit as well as treatment complications of the two treatment modalities.

## Supporting Information

S1 Protocol Study Protocol. (DOCX)

S1 CONSORT Checklist CONSORT Checklist. (TIF)

S1 Case Record Case Record. (DOCX)
