# Supplementary material for: Metformin versus Insulin in the Management of Pre-Gestational Diabetes Mellitus in Pregnancy and Gestational Diabetes Mellitus at the Korle Bu Teaching Hospital: A Randomized Clinical Trial
Source: PLoS One. 2015 May 6;10(5):e0125712. doi: 10.1371/journal.pone.0125712 (PMC4422739; doi:10.1371/journal.pone.0125712)
Supplement: S1 Protocol — (DOCX) [file pone.0125712.s001.docx]

### M.PHIL RESEARCH PROJECT PROPOSAL

**TITLE**

A study of metformin versus insulin in the management of gestational diabetes mellitus and type 2 pre-gestational diabetes mellitus at Korle Bu Teaching Hospital.

**PRINCIPAL INVESTIGATOR**

**Titus Beyuo**

**10084309**

Department of Pharmacology, University of Ghana Medical School

Email: captaint2003@yahoo.co.uk

Telephone: +233200284332

**DEGREE**

M. Phil Pharmacology

**DURATION**

June 2012-June 2013

**SUPERVISORS**

**Prof. Adjepon-Yamoah, (**Department of Pharmacology, UGMS)

**Prof. S. A. Obed,** (Dept. of Obstetrics and Gynaecology, UGMS)

**ABSTRACT**

**Background:** Diabetes mellitus is an important complication of pregnancy with adverse effects on both mother and fetus. Poor control of pre-gestational diabetes during the period of organogenesis increases the risk of major congenital malformations. While insulin is effective in controlling high blood glucose levels, otherwise resistant to diet and exercise management, several factors hinder its use. Metformin has been found to be a convenient, cheap, effective and safe hypoglycaemic agent in some countries and it is likely to do same among Ghanaian women.

**Aim:** The main aim of this study is to determine if metformin monotherapy or metformin in combination with insulin is equally effective at attaining and maintaining glycaemic targets compared to insulin monotherapy in the management of gestational diabetes mellitus and type 2 pre-gestational diabetes mellitus in Ghanaians.

**Methodology:**  A prospective randomized comparative study is proposed. Patient selection and randomization will be done using balloting with opaque envelopes. Participants will be followed through their index pregnancy with 2-weekly glycaemic profile monitoring and maternal weight gain. Babies delivered will be followed till the sixth week post-delivery measuring birth weight, incidence of birth trauma and neonatal intensive care unit (NICU) admission rates. Both laboratory and clinical data will be recorded and analyzed.

**Expected outcome:** It is expected that pregnant diabetics treated with metformin will have a lower 2HPG levels at the end of the study than those treated with insulin. The 2-hour post prandial glucose (2HPG) level is the measure selected to estimate the effectiveness of glycaemic control.

In addition NICU admissions are expected to be higher in the insulin group than metformin group.

**BACKGROUND**

Diabetes Mellitus is a metabolic disorder of multiple aetiology characterized by, chronic hyperglycaemia with disturbance of carbohydrate, fat and protein metabolism resulting from defects in insulin secretion, insulin action, or both ^1.^  Diabetes Mellitus is broadly grouped into Type I (Insulin Dependent Diabetes Mellitus) and Type II (Non- Insulin Dependent Diabetes Mellitus); the latter is the focus of this study. Diabetes Mellitus in pregnancy consists of pre-gestational Diabetes Mellitus and Gestational Diabetes Mellitus. Gestational Diabetes Mellitus is characterized by hyperglycaemia of varying severity, diagnosed during pregnancy (without previously known diabetes) and usually (but not always) resolving within six weeks of delivery^1^. The prevalence of diabetes mellitus world- wide is projected to increase.^2^

A study in Nigeria estimated the incidence of Diabetes Mellitus (DM) among pregnant women as 1.7%. Pre-gestational diabetes accounted for 39% while Gestational Diabetes was responsible for 61% of cases.^3^ There is no national data on the prevalence of DM among pregnant women in Ghana. Data from Greater Accra estimates the crude prevalence of Diabetes Mellitus as 6.3%^4^. It is estimated that 3-10 % of pregnancies are complicated by Diabetes Mellitus.^5, 6^

Diabetes mellitus is an important complication of pregnancy with adverse effects on both maternal and fetal outcomes. Treatment options of diabetes complicating pregnancy are limited. Several classes of medicines have been developed based on the pathophysiology of diabetes and are being used as monotherapy or combination therapies with varying efficacies in the general population of diabetics. In pregnant diabetics however, exercise and dietary management followed by insulin therapy where dietary management have failed is the general trend. Few countries have added some oral hypoglycaemic agents to this management, but lack of adequate data on efficacy and safety in the pregnant population has slowed this development in several countries.

The use of insulin has traditionally been the main stay in the management of DM in pregnancy not adequately controlled on diet and exercise. Though effective, the use of insulin is associated with some disadvantages such as the inconvenience of repeated injections, high cost, storage problems, hypoglycaemia, and fetal macrosomia amongst others. In one Indian study the cost of insulin was found to be ten-fold higher than metformin.^7^

A number of studies have examined the role of oral anti-diabetic medications in managing this condition in pregnancy. Metformin is one of the promising agents being widely used for this indication in many countries. It was first synthesized in the 1920s but was soon forgotten for the next two decades. Its history has been traced back to the use of a perennial herb, Galega officinalis in folklore medicine for the treatment of symptoms now known to be associated with DM.^11^

The first clinical trial of Metformin was performed by Sterne; He coined the name "Glucophage" (glucose eater) for the drug and published his results in 1957.^8^

Recent studies including randomized controlled studies suggest that metformin is more convenient, cheaper, effective and safe in pregnancy to mother and baby.^7, 9, 10^

**PROBLEM STATEMENT**

Diabetes Mellitus is a common disease in the adult population in both developed and developing countries. In developing countries in particular type 2 diabetes mellitus appears to be on the ascendancy because of life style changes and changing trends in dietary habits. As the disease burden increases in the general population it is expected that its prevalence in pregnancy will also increase. Pregnancies complicated by diabetes mellitus often require specialized care because of adverse maternal and fetal outcome if not properly managed. While several pharmacological options are available to diabetics in the general population for glycaemic control, the pregnant diabetic is often restricted to only insulin following dietary and exercise management failure. Insulin may not be affordable in low income countries. Frequent power outages or the absence of electricity also hinders its use in rural areas. In addition adherence to the frequent injections may be low.

Metformin taken orally is cheaper, more accessible and more user-friendly compared to insulin. Metformin is therefore a logical option for pregnant diabetics in Ghana. Recent clinical trials support metformin use in pregnancy, but lack of such data in Ghana still makes metformin use controversial. Its use during pregnancy has been hampered by lack of data on its efficacy and safety in pregnancy.

Another problem is that the pharmacogenetics of metformin does not support extrapolation of research findings from one place to another since its excretion is influenced significantly by a variant allele. Treatment failure or toxicity could occur in patients carrying the variant allele (*SLC22A2*).^16^

**HYPOTHESES**

- The glycaemic control will be equally effective with metformin therapy compared to insulin in pregnant diabetics in Ghana.
- Weight gain in mother and baby will be significantly lower on metformin therapy compared to insulin.

*Rationale:* Metformin has been shown to be equally effective and safe as insulin in the management of GDM. Poor compliance to insulin due to high cost, accessibility, storage difficulties and the discomfort of repeated injections is expected to make metformin a more suitable choice.

**AIM**

The aim of this study is to determine if metformin monotherapy or metformin in combination with insulin is more effective at attaining and maintaining glycaemic targets compared to insulin monotherapy in the management of gestational diabetes mellitus and type 2 pre-gestational diabetes mellitus in Ghanaians.

**SPECIFIC OBJECTIVES**

- To determine and compare blood glucose profile in pregnant women treated with metformin versus insulin.
- To determine the additional insulin requirements and the blood glucose profile in subjects who may require supplemental insulin in the metformin group.
- To measure and compare maternal weight gain in the index pregnancy between mothers treated with metformin versus insulin.
- To measure and compare birth weight between babies in the metformin and insulin groups.

**JUSTIFICATION**

Studies in countries like United States of America (USA), Australia, Canada and India have found metformin an effective and cheaper alternative to insulin. Genetic variations exist in the pathogenesis of diabetes mellitus as well as in the metabolism of metformin. Consequently results from one area may not be applicable in another. It is therefore important to do a Ghanaian study. There is no published data on the use of Metformin in pregnancy for glycaemic control in Ghanaians. The role of an effective but cheaper and more convenient to use medication in the control of any medical condition in a low – middle income country like Ghana cannot be overemphasized. Poor patient follow-up and monitoring in Ghana makes the use of drugs that do not require frequent monitoring, a preferred option at all times to those requiring frequent monitoring.

Insulin is less affordable and not readily available at all times. The need for refrigeration for effective insulin storage and use in developing countries is associated with difficulties. Unlike insulin, metformin does not require refrigeration for effective storage. Metformin is readily available and its oral administration is tolerable to many patients.

**LITERATURE REVIEW**

**Metformin**

Metformin is an oral hypoglycaemic agent which belongs to the biguanide class. Its history has been traced back to the use of a perennial herb, Galega officinalis. This herb has white, blue or purple flowers and grows over three feet high and is found in most temperate regions. Its common names include goat's rue, French lilac, Spanish sanfoin and false indigo. In folklore medicine, it is believed that G. officinalis was used to treat symptoms now ascribed to type 2 diabetes.^11^ G. officinalis continues to be cited for the treatment of diabetes in modern herbal pharmacopoeias.^11,12^ French physician Jean Sterne is credited with the first clinical trial of metformin (which he also called Glucophage) for the treatment of diabetes.^8, 11^

**CHEMISTRY/CLASSIFICATION**

The International Union of Pure and Applied Chemistry (IUPAC)’s systematic name for metformin is *N*,*N*-dimethylimidodicarbonimidic diamide**.** It is also referred to as 1,1-dimethylbiguanide**.** It is a basic compound with molecular mass 129.164 g/mol(free form) and 165.63g/mol (with HCl) .


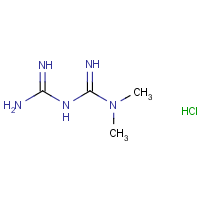

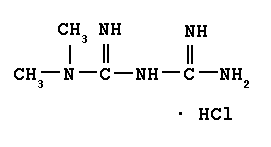


www.chemistrydaily.com

PHARMACOKINETICS

Metformin is administered orally and absorbed mainly in the small intestine with a bioavailability of 40-60% at doses of 0.5 to 1.0 g. Absorption is complete in 6 hours after oral administration. Its bioavailability tends to decrease with increasing doses. This inverse relationship has led to the proposal of an active, saturable absorption process.^12^  Its half-life is 2 hours. It is stable and does not bind to plasma proteins.^13, 14^ Metformin decreases absorption of vitamin B12 and folic acid, although reported cases of megaloblastic anemia are rare. Cimetidine decreases the elimination of metformin in healthy subjects. ^15^  Metabolism is insignificant and excretion is primarily renal.

The renal clearance of metformin in men and non-pregnant women has been found to correlate with creatinine clearance but exceeded glomerular filtration rate, indicating active net tubular secretion. ^13^

A parallel increase in renal clearance of both metformin and creatinine in mid and late pregnancy has been demonstrated. While metformin renal clearance increased on average by 49% and 29% in mid and late pregnancy respectively, compared with postpartum, the changes in renal clearance of creatinine were 29% and 21% respectively. The changes in metformin renal clearance were not dependent on the subjects OCT2 genotype ^19^ though metformin is known to be a substrate for organic cation transporters (OCTs). ^16, 17, 18^ Renal OCT2 (encoded by *SLC22A2*) has been suggested to play a significant role in the pharmacokinetics of metformin. This is because a 30 to 60% change in metformin secretion clearance and renal clearance and up to a 74% change in its area under the concentration-time curve (AUC) in carriers of the variant *SLC22A2* alleles has been noted. ^16, 20, 21^ Metformin has been shown to cross the placenta readily. Umbilical cord concentrations at the time of delivery are at least half of the maternal concentrations and in some cases even exceed them. ^22, 23, 24^ A study by Hebert, et al found umbilical cord plasma concentration of metformin to vary from undetectable (<5 ng/ml) to 1263 ng/ml at the time of delivery ^19^.

Infant exposure following metformin administration during breast-feeding has been found to be low. The relative infant dose is reported to be between 0.11 to 1.08% of the mother’s weight- adjusted dose ^22, 23, 24^.

**PHARMACODYNAMICS**

Metformin is an effective oral hypoglycemic agent that improves insulin sensitivity. ^25^ Metformin has a greater postprandial effect than the sulfonylureas and insulin, and is therefore more useful in patients with postprandial hyperglycaemia. The sulfonylureas and insulin on the other hand are more effective in managing poorly controlled fasting hyperglycaemia. ^15^
 Metformin reduces free testosterone levels and increased sex hormone-binding globulin (SHBG). It improves menstrual irregularities leading to spontaneous ovulation and improved ovarian response to conventional ovulation induction therapies. ^25,^

The introduction of metformin for the treatment of polycystic ovary syndrome (PCOS) provided evidence supporting its use in pregnant women. ^28^ Metformin has been recommended for use during pregnancy for the treatment of gestational as well as preexisting diabetes mellitus. ^29^

The exact mechanism of action of Metformin is not fully understood despite its wide clinical usage. Metformin does not stimulate insulin secretion.
It exerts its hypoglycaemic effect via metabolic activities at several sites of action, including liver, adipocytes, intestine, and muscle cells. The activities in these sites of action (Biophases) include:

1) Decreased hepatic glucose output due to decreased hepatic gluconeogenesis and increased glycogenesis and lipogenesis; ^30^

2) Reduced rate of intestinal glucose absorption; ^31^ and

3) Increased glucose uptake by muscle cells and adipocytes ^32^

The relative contribution of these individual sites of action to the overall glucose-lowering effect is unknown and a subject of continuing research in animal models^33^. This is because the dose-response relationship of the metabolic effects in individual organs and tissues is obscured by the multifactorial mechanism of metformin and the complex nature of glucose homeostasis in-vivo ^33^

**METHODOLOGY**

STUDY DESIGN

The study design is a prospective, randomized comparative study. Participants will be randomly assigned into one of the two treatment groups. For a set of four (4) patients seen at the clinic for the first time, they will be made to ballot by picking randomly one paper with an inscription each from an opaque envelope. This assigned participants to one of the two treatment groups – insulin and metformin. This will be repeated till the sample size is attained. The sequence of picking will be in the order in which they reported to the clinic; “first to report, first to pick”**.**  The participants in each group will be matched for age, parity, gestational age, pre-treatment weight and pre-treatment glycaemic profile.

Treatment will be administered to each according to the study treatment protocols. Subjects in both groups will follow the routine antenatal schedule and their blood glucose levels will also be checked and recorded during these visits according to existing protocols. For in-patients a similar routine will be followed.

STUDY AREA

The study will be conducted at the Maternity Unit and the Diabetes Centre of Korle Bu Teaching Hospital. It will involve both in- patients and out-patients in these units.

STUDY SUBJECTS

**Eligibility criteria**

Eligible subjects will be women aged 18 to 45 years who are pregnant with single fetus at gestational age of 20 to 30 weeks and have been diagnosed with type 2 DM or Gestational Diabetes and met the Hospital’s criteria for starting insulin therapy. The criteria for the diagnosis of GDM and DM will be a plasma glucose concentration of greater than or equal to 7 mmol/L after an overnight fast or plasma glucose concentration of equal to or greater than 11.1 mmol/L 2 hours after a 75g glucose drink.^1^

The current criteria for starting insulin therapy in patient with GDM at the diabetes clinic of KBTH are at least one following:

- FBS > 5.1 mmol/l
- 1HPG > 10.0 mmol/L or
- 2HPG > 8.5 mmol/L.

Subjects diagnosed according to the above criteria that are unable to achieve the above targets following management on diet and exercise will be eligible for the study.

**Exclusion criteria**

Exclusion criteria will include patients with insulin dependent Diabetes Mellitus and patients with contraindications to metformin therapy.

SAMPLE SIZE DETERMINATION

Power calculation: The power calculation is based on the difference in 2HPG expected between the metformin and insulin groups at 34 weeks gestation. Using the two sampled mean formula^34^, the estimated sample size was 47 per group. From pre-trial estimates the minimum difference in mean 2HPG levels between the two groups of 4mg/dl (0. 22 mmol/l) is required to give a power of 80% to the study at a significance level of α = 0.05.We recruited 52 in each group to allow for a 10% non-respondent rate

TREATMENT PROTOCOL

**Metformin Group**

In the Metformin group, starting dose of Metformin will be 500mg once a day and increased gradually over two (2) weeks, to meet glycaemic targets of FBS < 5.1 mmol/L, 1HPG < 10.0mmol/L and 2HPG < 8.5 mmol/L.

The maximum dose will be 2500mg per day. Insulin will be added if targets cannot be reached on metformin alone at maximum doses. Subjects that receive supplemental insulin will be excluded from the metformin group and analyzed for the purpose of this study as treatment failures. The quantity of supplemental insulin will be documented for analysis.

**Insulin group**

In the Insulin group both soluble insulin and premixed insulin will be prescribed. There will be no brand restriction. Both premixed insulin and soluble insulin will be administered subcutaneously in the deltoid region. Total daily dose of premixed insulin at initiation will be calculated for most patients as 0.3 IU/kg body weight. However for patients admitted with high blood glucose levels they will be managed on sliding scale with soluble insulin their starting dose will be based on total daily requirement. The total daily dose will then be divided into two: two-thirds of the dose will be administered in the morning 30 minutes before breakfast and one-third of the daily dose will be administered in the evening 30 minutes before supper. The total dose of insulin will be titrated for each patient to achieve the above glycemic targets. Patients who may not achieve glycemic targets on their out-patient doses after attempts at titrations will be admitted to the ward and treated with soluble insulin to determine their new optimum insulin requirements. All patients will be educated by both nurses and doctors while on admission on the disease and self-administration of the correct doses of insulin before discharge.

**Treatment failures**

Supplemental insulin for treatment failures in the Metformin group will be prescribed according to the same protocols in the insulin group.

DATA COLLECTION

Basic demographic data will be recorded. They will be followed through their index pregnancy, with 2-weekly blood glucose checks in hospital. All laboratory samples will be collected and analyzed at the diabetes research laboratory by staff of the same laboratory. Venous blood will be used for blood glucose profile analysis. Analysis of Fasting Blood Glucose (FBG), one- hour post-prandial glucose (1HPG), two- hour post-prandial glucose (2HPG) will be done using the Mindray BS-400^®^ (Mindray Medical Int. Ltd, China) chemistry analyzer. The method for glucose determination will be enzymatic photometric testing. Urinalysis, weight records (using Seca^®^(gmbh&Co. Kg Germany)) and cost of treatment will also be documented. The optimal dose of metformin required for optimal glycaemic control will be recorded for each patient. Peri-partum events like gestational age at delivery, type of delivery, fetal birth weight, and NICU admissions will be retrieved from patient notes.

STATISTICAL ANALYSIS

In analyzing the data, summary statistics will include appropriate tables and charts. Categorical data will be represented by pie and bar graphs and continuous data with histograms.

For inferential statistics, significant difference of mean observations using one-tail student’s t-test and z-score for differences in proportions where applicable. The differences in trends of glycaemic profile over time between the study groups will be assessed by two-way repeated measures analysis of variance (ANOVA).

Significance level (p-value) will be set at α = 0.05. Data analysis will be done using statistical package for social science **(SPSS** version 20).

**EXPECTED OUTCOME**

It is expected that pregnant diabetics treated with metformin will have a lower 2HPG levels at the end of the study than those treated with insulin. The primary outcome is the difference in the 2HPG level between the metformin and insulin group. The rationale for selecting 2HPG is that by its mechanism of action, metformin is expected to have a more significant blood glucose lowering effect in the postprandial period than the fasting period. Hyperglycaemia in the prandial period is related to insufficient suppression of hepatic glucose output, poor insulin release and ineffective glucose disposal in target tissues (especially muscles) by insulin (insulin resistance) and metformin affect all these Biophases.^30-32^

Secondary outcomes to be measured include glycaemic profile (measured by FBG, 1HPG, and HB1AC), fetal birth weight, maternal weight gain and caesarean section rate which are expected to be lower in the metformin group, number of NICU admission which is expected to be higher in the insulin group. If found to be effective it may form the basis for developing a policy for Diabetes Mellitus care in pregnancy at primary and secondary level of healthcare instead of the current tertiary level of care.

**BENEFICIARIES**

MOH, GHS, Diabetes services, Diabetologist; Obstetricians and diabetes healthcare providers as well as all women whose pregnancies maybe complicated by diabetes mellitus.

**DISSEMINATION OF RESULTS**

Results from the study will be presented to the department of pharmacology, University of Ghana Medical School at the M. Phil seminars I & II. It will also be compiled into a thesis and presented to the School of Graduate studies, University of Ghana. Copies will be distributed to various libraries. Findings will also be published in a recognized scientific journal.

**ETHICAL CONSIDERATIONS**

A written informed consent will be sought from all participants. Confidentiality of participants’ data will be assured. Standard protocols for clinical research involving human subjects will be strictly observed.

Data collected will be used for purely research purposes and completed questionnaire will be destroyed upon successful completion of the study.

This proposal will be submitted to Ethical and Protocol Review Committee of University of Ghana Medical School for review and approval.

**BUDGET**

| **ITEM** | **COST (GH ¢)** |
| --- | --- |
| Supplies, Reagents and consumables | **1500** |
| Transportation | **1000** |
| Printing and binding | **200** |
| Stationery | **100** |
| **TOTAL** | **2, 800** |

**ACTIVITY SCHEDULE**

| ACTIVITIES | DURATION: MAY 2012 –MAY 2013 | | | | | | | | | | | | |
| --- | --- | --- | --- | --- | --- | --- | --- | --- | --- | --- | --- | --- | --- |
|  | **May** | **Jun** | **Jul** | **Aug** | **Sep** | **Oct** | **Nov** | **Dec** | **Jan** | **Feb** | **Mar** | **Apr** | **May** |
| Proposal  Writing | * | * | * | * | * | * |  |  |  |  |  |  |  |
| Proposal Presentation at Pham Dept. |  | * |  |  |  |  |  |  |  |  |  |  |  |
| Preliminary workAcquisition of Materials and recruiting participants |  |  | * | * | * |  |  |  |  |  |  |  |  |
| Actual work of participant follow-up and data collection,  Processing/Analysis |  |  |  | * | * | * | * | * | * | * |  |  |  |
| Data Analysis |  |  |  |  |  |  |  | * | * | * |  |  |  |
| Write up | * | * | * | * | * | * | * | * | * | * | * | * |  |
| Presentation of  Research Findings to Pham Dept. |  |  |  |  |  |  |  |  |  |  |  | * |  |
| Submission of  Thesis |  |  |  |  |  |  |  |  |  |  |  |  | * |

**REFERENCES**

1. World Health Organization (WHO) Department of Noncommunicable Disease Surveillance. *Definition, diagnosis and classification of diabetes mellitus and its complications. Report of a WHO consultation. Part 1: diagnosis and classification of diabetes mellitus*. Geneva: WHO, *1999*.
2. Ben-Haroush A, Yogev Y, Hod M. Epidemiology of gestational diabetes mellitus and its association with Type 2 diabetes. Diabet Med 2004;21:103-113
3. Ozumba BC, Obi SN, Oli JM. Diabetes mellitus in pregnancy in an African population. *Int J Gynaecol Obstet.*2004;84(2):114–119.
4. Amoah AGB, Owusu [SK](http://www.diabetesresearchclinicalpractice.com/article/S0168-8227(01)00374-6/abstract), Adjei S. Diabetes in Ghana: a community based prevalence study in Greater AccraDiabetes *Research and Clinical Practice* 2002; 56(3):197-205
5. Thorpe LE, Berger D, Ellis JA, Bettegowda VR, Brown G, Matte T, Basset M, Frieden TR. Trends and racial/ethnic disparities in gestational diabetes among pregnant women in New York city, 1990–2001. *Am J Pub Hlth.*2005;95(5):1536–1539.
6. Ferrara A, Hedderson MM, Selby JV. Prevalence of gestational diabetes mellitus detected by the National Diabetes Data group or the Carpenter and Coustan plasma glucose thresholds. *Diabetes Care.*2002;25(9):1625–1630.
7. Rai L, [Meenakshi D](http://www.ncbi.nlm.nih.gov/pubmed?term=%22Meenakshi%20D%22%5BAuthor%5D), [Kamath A](http://www.ncbi.nlm.nih.gov/pubmed?term=%22Kamath%20A%22%5BAuthor%5D). [Metformin--a convenient alternative to insulin for Indian women with diabetes in pregnancy *J Med Sci*.](http://www.ncbi.nlm.nih.gov/pubmed/20075550) 2009 Nov;63(11):491-7.
8. Campbell IW. Metformin—life begins at 50: A symposium held on the occasion of the 43rd Annual Meeting of the European Association for the Study of Diabetes, Amsterdam, The Netherlands, September 2007. *The British Journal of Diabetes & Vascular Disease*. 2007;7:247–252.
9. Moore LE, Briery CM, Clokey D, Martin RW, Williford NJ, Bofill JA, Morrison JC Metformin and insulin in the management of gestational diabetes mellitus: preliminary results of a comparison.*. J Reprod Med.* 2007 Nov; 52(11):1011-5*.*
10. Rowan JA., Hague WM., Gao W, Battin MR., Moore MP. Metformin versus Insulin for the Treatment of Gestational Diabetes *N Engl J Med* 2008;358:2003-15.
11. Bailey CJ, Day C. [Metformin: its botanical background](http://www3.interscience.wiley.com/cgi-bin/fulltext/108564133/HTMLSTART). *Practical Diabetes International*. 2004;21(3):115–7.
12. Duke JA. Handbook of medicinal herbs, 2nd edn. Boca Raton, Florida: CRC Press, 2002; 337).
13. Scheen AJ Clinical pharmacokinetics of metformin*. Clin Pharmacokinet* 1996; 30:359–371.
14. Hardman, J.G., L.E. Limbird, P.B., A.G. Gilman. Goodman and Gilman's The Pharmacological Basis of Therapeutics. 10th ed. New York, NY: McGraw-Hill, 2001., p. 1705
15. Melchior WR, Jaber LA Metformin: an antihyperglycemic agent for treatment of type II *diabetes.*Ann Pharmacother 1996; 30 (2): 158-164.
16. Wang ZJ, Yin OQ, Tomlinson B, Chow MS OCT2 polymorphisms and in-vivo renal functional consequence: studies with metformin and cimetidine*. Pharmacogenet Genomics* 2008; *18:637–645.*
17. Kimura N, Okuda M, Inui K Metformin transport by renal basolateral organic cation transporter hOCT2. *Pharm Res* 2005; 22:255–259
18. Zhou M, Xia L, Wang J Metformin transport by a newly cloned proton-stimulated organic cation transporter (plasma membrane monoamine transporter) expressed in human intestine. *Drug Metab Dispos* 2007 *;*35:1956–1962*.*
19. Hebert MF, Eyal S, Easterling TR, Carr D, Umans JG, Miodovnik M, Hankins GDV, Clark SM, Risler L, Wang J, Kelly EJ, Shen DD. Pharmacokinetics of Metformin during Pregnancy *DMD* 2010: 38(5) 833-840
20. Chen Y, Li S, Brown C, Cheatham S, Castro RA, Leabman MK, Urban TJ, Chen L,Yee SW, Choi JH, et alEffect of genetic variation in the organic cation transporter 2 on the renal elimination of metformin. *Pharmacogenet Genomics* 2009; 19:497–504*.*
21. Song IS, Shin HJ, Shim EJ, Jung IS, Kim WY, Shon JH, Shin JG *(2008) Genetic variants of the organic cation transporter 2 influence the disposition of metformin. Clin Pharmacol Ther 84:559–562.*
22. Hague WM, Davoren PM, McIntyre D, Norris R, Xiaonian X, Charles BMetformin crosses the placenta: a modular for fetal insulin resistance (Letter)? *Br Med J* 2003; 327:880–881.
23. Vanky E, Zahlsen K, Spigset O, Carlsen SM Placental passage of metformin in women with polycystic ovary syndrome. *Fertil Steril* 2005; 83:1575–1578*.*
24. Charles B, Norris R, Xiao X, Hague W Population pharmacokinetics of metformin in late pregnancy*. Ther Drug Monit* 2006; 28:67–72*.*
25. Krentz AJ, Bailey CJ Oral antidiabetic agents: current role in type 2 diabetes mellitus. *Drugs* 2005; 65:385–411*.*
26. Awartani KA, Cheung AP Metformin and polycystic ovary syndrome: a literature review J Obstet Gynaecol Can 2002 ; 24 (5): 393-401
27. De Sloover KY, Ernst ME Use of metformin in polycystic ovary syndrome Ann Pharmacother 2001; 35 (12): 1644-1647
28. Lord JM, Flight IH, Norman RJ *Metformin in polycystic ovary syndrome: systematic review and meta-analysis. BMJ* 2003; 327:951–953*.*
29. Guideline Development Group (2008) Management of diabetes from preconception to the postnatal period: summary of NICE guidance. BMJ 2008; 336:714–717*.*
30. Christiansen MP, Hellerstein MK Effects of metformin on hepatic glucose metabolism*. Curr Opin Endocrinol Diabetes* 1998; 5:252–255*.*
31. Wilcock C, Bailey CJ Reconsideration of inhibitory effect of metformin on intestinal glucose absorption*. J Pharm Pharmacol* 1991; 43:120–121.
32. Bailey C, Path M, Turner M Metformin*. N Engl J Med* 1996; 334:574–579*.*
33. Kuhlmann J, Puls W, Wiernsperger N *(1996)* Preclinical pharmacology of biguanides in: *Oral Antidiabetics, (Springer Verlag, Berlin).*
34. Chow SC, Shao J and Wang H.( 2002) A note on sample size calculation for mean comparisons based on non-central T-Statistics*. J. Biopharm. Stat*, 12 (4): 441–456.

**CONSENT FORM**

Dear participant,

We are undertaking a study to compare the use of metformin versus insulin in managing diabetes mellitus in pregnancy. I would appreciate it if you would volunteer to be part of this study.

Kindly read the information below and give consent to be part of this study, if you agree. Thank you,

Dr. Titus Beyuo (Principal Investigator)

(0200284332)

Information for participants

1. Participation is not compulsory
2. You can withdraw from the study at any time if you are not comfortable
3. This study has been designed to ensure your safety as well as that of your baby. Therefore your participation will not endanger your life or your baby’s life.
4. You will still receive the best of care from your doctors if you don’t participate or decide to withdraw later form this study.
5. Information and samples (blood, urine, breast milk etc.) taken from you will be used only for the purpose of this study.
6. Strict confidentiality will be maintained and your identity will not be disclosed throughout this study, or when findings of this study are presented at any forum print or electronic.

I ………………………………………………………………………………., have read and understood the above information / have understood the above information as explained to me. All questions on the above information or the study, not clear to me have also been explained to my understanding in a language I understand. I therefore give my consent to participate in this study.

……………………………………………. …………………………..

Participant (signature/RTP) Investigator (Signature)

Date……………………………………
