# Supplementary figures and images for: Metformin versus Insulin in the Management of Pre-Gestational Diabetes Mellitus in Pregnancy and Gestational Diabetes Mellitus at the Korle Bu Teaching Hospital: A Randomized Clinical Trial
Source: PLoS One. 2015 May 6;10(5):e0125712. doi: 10.1371/journal.pone.0125712 (PMC4422739; doi:10.1371/journal.pone.0125712)

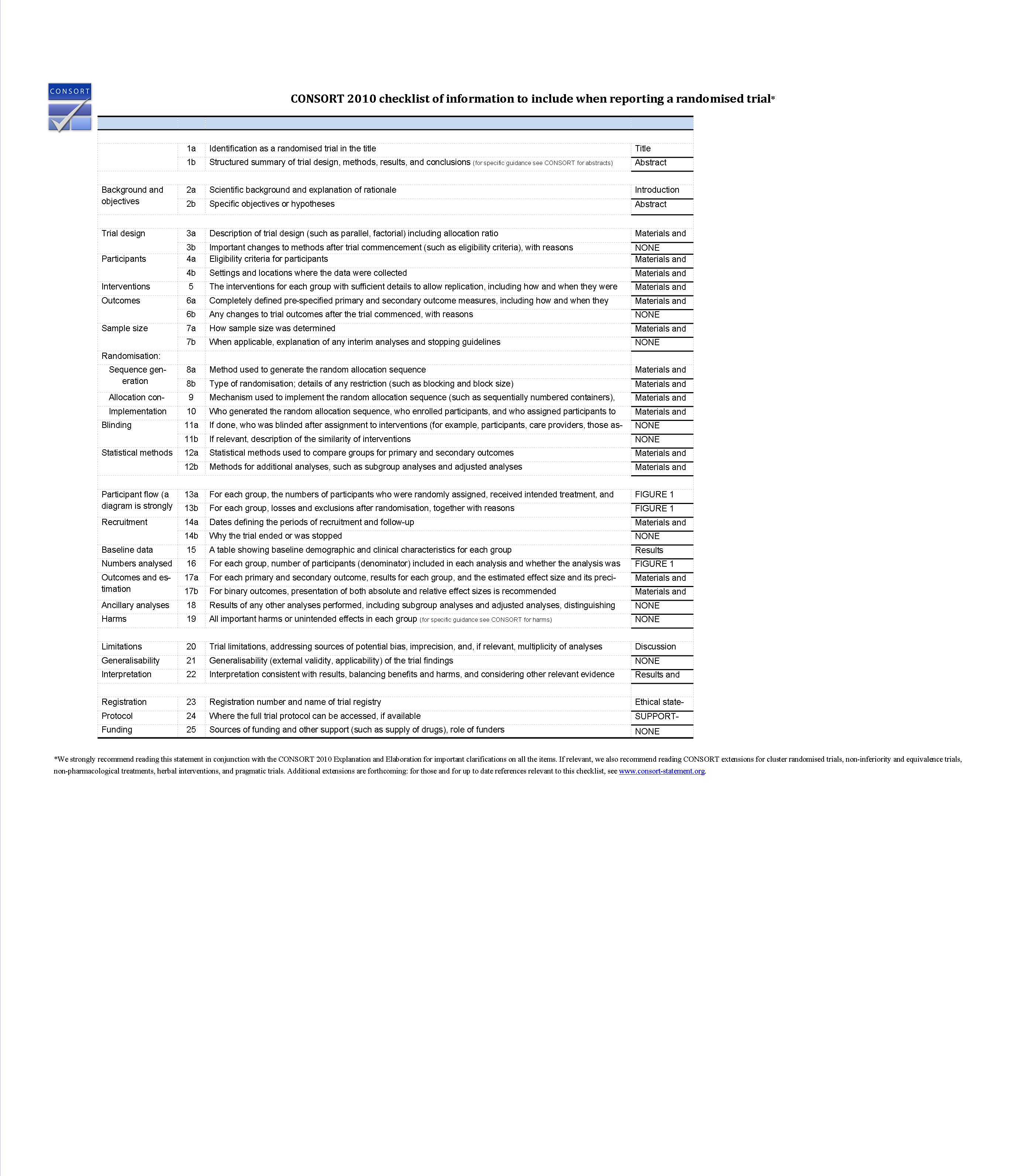

Supplement: S1 CONSORT Checklist — (TIF) [file pone.0125712.s002.tif]
