# Supplementary material for: Metformin versus Insulin in the Management of Pre-Gestational Diabetes Mellitus in Pregnancy and Gestational Diabetes Mellitus at the Korle Bu Teaching Hospital: A Randomized Clinical Trial
Source: PLoS One. 2015 May 6;10(5):e0125712. doi: 10.1371/journal.pone.0125712 (PMC4422739; doi:10.1371/journal.pone.0125712)
Supplement: S1 Case Record — (DOCX) [file pone.0125712.s003.docx]

**CASE RECORD**

**A study of metformin versus insulin in the management of gestational diabetes mellitus and type 2 pre-gestational diabetes mellitus at Korle Bu Teaching Hospital.**

Date enrolled: __ __/__ __/20__ __ Time:__ __/__ __ hrs Folder No:___________________

Randomization group: __________________________________________________________

Telephone No._________________________________________________________________

Name: _______________________________________________ Gravidity [ __ ] Parity [ __ ]

Date of birth……………………………………………………………... __ __/__ __/__ __ __ __

Age (last half year)………………………………………………………....years [ __ ] mths [ __ ]

Marital status (1=married, 2= single/divorced/separated/widowed)………………………… [ __ ]

Source of financial support for index pregnancy (1= self only, 2= partner only, 3= self and partner, 4= others) ……………………………………………………………………..……. [ __ ]

Weight at enrollment (kg) ……………………………………………….. [ __ ] [ __ ] [ __ ].[ __ ]

Height (meters) …………………………………………………………..……….. [ __ __ ].[ __ ]

LMP…………………………………………………………………………..__ __/__ __/20__ __

EDD (by LMP)…………………………………………………………….…__ __/__ __/20__ __

EDD (by early/first ultrasound scan)……………………………………...…__ __/__ __/20 __ __

Gestational age (by LMP estimate)………………………………….weeks [ __ __ ]days[ __ __ ]

Gestational age (by ultrasound scan estimate) …………………...…weeks [ __ __ ] days[ __ __ ]

Classification of diabetes mellitus (1=GDM, 2=T2DM)……………………………………. [ __ ]

Co-morbid conditions in this (index) pregnancy (please tick all that apply)

1. Pregnancy induced hypertension (PIH)………………………………………...……. [ __ ]
2. Essential hypertension ………………………………………………………………. [ __ ]
3. Sickle cell disease ………………………………………………….………………... [ __ ]
4. Asthma …………………………………………………………..………………….. [ __ ]
5. Others (specify) __________________________________________________________

Obstetric history [previous pregnancies/deliveries] (please tick all that apply)

1. Miscarriage(s)………………………………………………………………………... [ __ ]
2. Stillbirth(s) ……………………………………………………………….…………. [ __ ]
3. Early neonatal death(s) ……………………………………………………...………. [ __ ]
4. Big (Macrosomic) baby (birth weight greater 4.5kg) ……..……………………...… [ __ ]
5. Congenital anomaly …………………………………………………………….…… [ __ ]
   - Specify ___________________________________________________________
6. Caesarean section……………………………………………………………………. [ __ ]
7. Other(s) specify __________________________________________________________

Baseline measurements

1. FBG (mmol/L) ……………………………………………...…………. [ __ ] [ __ ]. [ __ ]
2. 1HPG (mmol/L)……………………………………………...………… [ __ ] [ __ ]. [ __ ]
3. 2HPG (mmol/L) …………………………………………...…………... [ __ ] [ __ ]. [ __ ]
4. Maternal weight (kg) …..…………..…………………………..… [ __ ] [ __ ] [ __ ].[ __ ]

Measurements at one month

1. FBG (mmol/l) ……………………………………………...…………. [ __ ] [ __ ]. [ __ ]
2. 1HPG (mmol/L)……………………………………………...………… [ __ ] [ __ ]. [ __ ]
3. 2HPG (mmol/L) …………………………………………...…………... [ __ ] [ __ ]. [ __ ]
4. Maternal weight (kg) …..…………..…………………………..… [ __ ] [ __ ] [ __ ].[ __ ]

Measurements at 36/37 weeks gestation

1. FBG (mmol/L) ……………………………………………...…………. [ __ ] [ __ ]. [ __ ]
2. 1HPG (mmol/L)……………………………………………...………… [ __ ] [ __ ]. [ __ ]
3. 2HPG (mmol/L) …………………………………………...…………... [ __ ] [ __ ]. [ __ ]
4. Maternal weight (kg) …..…………..…………………………..… [ __ ] [ __ ] [ __ ].[ __ ]

**Maternal peri-partum events**

Mode of delivery (1=SVD, 2=Assisted vaginal delivery, 3= C/S)………………………..… [ **__ ]**

If Caesarean section what is the indication: 1.____________________________________________________________________________2.____________________________________________________________________________3.____________________________________________________________________________

**Neonatal peri-partum events**

Gestational age at delivery ………………………………………...…. weeks [ __ __ ] days [ __ ]

Outcome of delivery (1 =live birth,2 =fresh stillbirth, 3= macerated stillbirth) …………….[ __ ]

Birth weight (kg)……………………………………………….…….….. [ __ ] [ __ ] [ __ ].[ __ ]

APGAR score ………………………………………...………………….. 1 min [ __ ]5min [__ ]

Birth trauma ^*^ (1=none, 2=mild, 3=moderate/severe)…………………………….… [ __ ]

Was baby resuscitated? (1= yes, 2= no) …………….………………..……………. [__ ]

NICU referral (1= yes, 2 = no) ….………….……...………………...……………… [ __ ]

If yes, reason for referral (please tick all that apply)

1. Pre-maturity ………………………………………………………...………… [ __]
2. Birth asphysia ……………………………………………………..………….. [ __]
3. Macrosomia (big baby)…………………………………………………….…. [__ ]
4. Respiratory distress syndrome………………………………………………... [__ ]
5. Birth trauma………………………………………………………………...… [__ ]
6. Congenital anomalies……………………………………………………….… [__]
7. Neonatal jaundice…………………………………………………………..… [__ ]
8. Meconium aspiration…………………………………………………….…… [__ ]
9. Sepsis /at risk of sepsis………………………………….………………..…… [__]
10. Small for gestational age (SGA) (BW less than 2.5kg)………………………. [__ ]
11. Others (specify) _______________________________________

**Complete this portion for babies seen at NICU only.**

NICU Admission (1= yes, 2= no) …………………………………………………... [__]

Duration of admission (days) ……………………………………………….… [__ __ __ ]

Diagnosis at NICU (tick all that apply)

1. Pre-maturity ………………………………………………………...………… [ __]
2. Birth asphysia ……………………………………………………..………….. [ __]
3. Macrosomia (big baby)…………………………………………………….…. [__ ]
4. Hypoglycaemia (less than 2.6mmol/l) …………………………………...….. [__ ]
5. Respiratory distress syndrome………………………………………………... [__ ]
6. Birth trauma………………………………………………………………...… [__ ]
7. Congenital anomalies……………………………………………………….… [__]
8. Neonatal jaundice…………………………………………………………..… [__ ]
9. Meconium aspiration…………………………………………………….…… [__ ]
10. Sepsis /at risk of sepsis………………………………….………………..…… [__]
11. Small for gestational age (BW less than 2.5kg)………………………………. [__ ]
12. Others (specify) _______________________________________

Outcome of admission (1=discharged; 2= died) ……………………………………………. [ __ ]

**ADVERSE EVENTS**

**Maternal** (tick all that apply)

1. Antenatal admissions ………………………………………………………………... [ __ ]

Number of admissions ………………………………………………………………. [ __ ]

If admitted, reasons for admission;

Glycaemic control ……………….………………………………………………….. [ __ ]

BP control …………………………………………………………………………… [ __ ]

Premature rupture of membranes …………………………………………….…..…. [ __ ]

Bleeding per vagina (specify cause) __________________________________________

Others (specify)___________________________________________________________

1. Gastrointestinal events requiring dose reduction……………………………..…..…. [ __ ]
2. Gastrointestinal events requiring treatment cessation…………………………...….. [ __ ]
3. Renal impairment (mild/moderate) ………………….…………………….………. [ __ ]
4. Renal impairment (severe requiring dialysis) …………………………………….… [ __ ]
5. Surgery other than C/S (specify with indication) …………………………………………

……………………………………………………………………………………………..…

1. Maternal death ……………………………………………………………………… [ __ ]

**Fetal/neonatal**

1. Intrauterine growth restriction (confirmed by ultrasound) ………….………….….. [ __ ]
2. Fetal distress ……………………..……………………………………………..….. [ __ ]
3. Intrauterine fetal death ………………………………........... ……………….…… [ __ ]

State gestational age at which diagnosis was made..… weeks [ __ __ ]days [ __ ]

1. Other events (specify) _____________________________________________________

**2 WEEKLY MONITORING**

| DATE | GESTATION (WEEKS) | WEIGHT  (kg) | FBG  (mmol/L) | 1HPG  (mmol/L) | 2HPG  (mmol/L) |
| --- | --- | --- | --- | --- | --- |
|  |  |  |  |  |  |
|  |  |  |  |  |  |
|  |  |  |  |  |  |
|  |  |  |  |  |  |
|  |  |  |  |  |  |
|  |  |  |  |  |  |
|  |  |  |  |  |  |
|  |  |  |  |  |  |
|  |  |  |  |  |  |
|  |  |  |  |  |  |
|  |  |  |  |  |  |

**TREATMENT PROTOCOLS**

**Treatment targets**

1. FBS < 5.5 mmol/l
2. 2HPG < 7.0 mmol/L.

**Metformin Group**

1. Starting dose of Metformin will be 500mg once a day
2. Increased by 500mg every two (2) weeks, to meet glycaemic targets.
3. Maximum dose should not exceed 2500mg per day.
4. Please add Insulin on reaching maximum dose according to insulin protocol below. Please note that maximum tolerable dose of each patient may differ from the stated maximum dose..

**Insulin group**

1. Starting dose is 0.3 international units per kilogramme body weight.
2. Divide the total dose calculated above into two.
3. Give 2/3 of the dose in the morning 30 minutes before breakfast and 1/3 of the daily dose in the evening 30 minutes before supper.
4. Administer subcutaneously in the deltoid region.
5. Adjust daily dose for each patient to achieve the above glycaemic targets.
6. The maximum dose of premixed insulin for out-patient care for the purpose of this study is 1.0 international units per kilogramme body weight
7. Please refer patients who are unable to achieve targets after titrating to maximum doses for admission to the maternity ward.
8. Treat the above patients (in point 7) with soluble insulin to determine their optimum insulin requirements. When their glycaemic targets are met, premixed insulin dose equivalent to the total soluble insulin requirements per day while on admission can be started before discharge.

**Treatment failures**

1. Supplemental insulin for treatment failures in the Metformin group should be prescribed according to the same protocols in the insulin group.
